# Effects of obesity and hormone therapy on surgically-confirmed fibroids in postmenopausal women

**DOI:** 10.1007/s10654-015-0016-7

**Published:** 2015-03-18

**Authors:** Eva M. Sommer, Angela Balkwill, Gillian Reeves, Jane Green, Dame Valerie Beral, Kate Coffey

**Affiliations:** Cancer Epidemiology Unit, University of Oxford, Richard Doll Building, Roosevelt Drive, Old Road Campus, Oxford, OX3 7LF UK

**Keywords:** Uterine leiomyoma, Fibroids, Postmenopausal, BMI, HRT, Million Women Study

## Abstract

To examine the association between body mass index (BMI), use of menopausal hormone therapy (HT), and incidence of uterine fibroids in postmenopausal women, 610,604 postmenopausal women without prior hysterectomy or diagnosis of fibroids were followed as part of a large United Kingdom prospective cohort study. We used Cox regression models to calculate adjusted relative risks (RRs) of surgically-confirmed fibroids (defined as a hospital admission with uterine fibroids as a primary diagnosis with a related surgical procedure), in relation to BMI and use of HT. During an average of 11.4 years of follow-up, 3561 women were admitted to hospital with surgically-confirmed fibroids. Five-year incidence rates decreased with age, from 0.50 % (1 in 200 women) at age 50–54, to 0.11 % (1 in 1000 women) at age 75–79. The 5-year rate in postmenopausal women aged 50–54 was about a quarter that seen in premenopausal women of the same age (1 in 200 vs. 1 in 50). Compared with normal weight women, obese women had a RR of surgically-detected fibroids of 1.46 (95 % CI 1.33–1.59; *p* < 0.0001). HT use was associated with a RR of 2.33 (95 % CI 2.18–2.49; *p* < 0.0001) in ever versus never users. When we analysed HT use and BMI together, obese vs. normal weight never users had a RR of 2.00 (95 % CI 1.77–2.26): the highest risks were seen in women who were obese and had ever used HT, RR = 3.30 (95 % CI 2.88–3.79). Uterine fibroids continue to occur in postmenopausal women; obesity and hormone therapy use are important modifiable risk factors.

## Introduction

Uterine fibroids are benign monoclonal smooth muscle tumours of the uterus [1], and are the most common pelvic tumour in women [2]. Asymptomatic in at least 50 % of cases [3], fibroids are nonetheless an important cause of morbidity and a common reason for surgery. A recent study estimated the annual cost of treating uterine fibroids in the United States at $34.4 billion dollars [4].

Prevalence rates ranging from 5 to 77 % [5] have been reported, reflecting differences in case definition and population studied. The highest estimates come from pathologic examination of hysterectomy specimens [6]. Fibroids are an indication for a large proportion of hysterectomies [7]; estimates based on pathological findings are likely to overestimate prevalence, and may preferentially identify women with specific symptoms, such as pain or bleeding [8]. Reported risk factors for uterine fibroids include black ethnicity, family history, parity, and obesity [1, 2, 9–11].

Factors that increase exposure to oestrogen appear to increase the incidence of uterine fibroids [10]. Fibroids are not seen before puberty, occur most commonly in women of reproductive age, and are commonly said to regress after the menopause [12]. The reduction in size or resolution of fibroids in postmenopausal women [10–12] is thought to be due to the lower average levels of endogenous ovarian hormones after the menopause. However, in postmenopausal women average oestrogen levels have been found to increase with increasing body mass index (BMI) [13]. Use of exogenous hormones in the form of menopausal hormone therapy (HT) might also be expected to increase risk, although evidence for this has been relatively scarce [12].

The Million Women Study provides a unique opportunity to examine the relationship between adiposity, HT use and the incidence of uterine fibroids in postmenopausal women. This prospective cohort study collected detailed information at recruitment on participants’ menopausal status, use of HT, and height and weight. There is virtually complete follow-up for cause-specific hospital admissions, incident cancers and death.

In order to clarify the public health impact of fibroids in postmenopausal women, we used a strict case definition, including only clinically relevant disease involving surgical detection and/or treatment. We aimed to determine whether postmenopausal women who were overweight or obese were more likely to have surgically-confirmed fibroids than normal weight women. We also investigated whether women who had ever used HT were at an increased risk of fibroids, and whether the use of HT modified the association with BMI.

We have used the term ‘incidence rate’ throughout the study, however surgically-confirmed fibroids in postmenopausal women represent a subset of true incidence; cases in our analysis are those in which uterine fibroids, or a co-morbid condition, have resulted in a surgical diagnosis of fibroids.

## Materials and methods

The Million Women Study is a large prospective cohort study which recruited 1.3 million women, most aged 50–64 years, between 1996 and 2001 via the National Health Service (NHS) Breast Screening Programme. Full details of the study design and methods are described elsewhere [14]. Approximately one in two women in this age range in the recruitment areas agreed to participate in the study, or around one in four women in this age range in the entire United Kingdom. Participants gave written informed consent for use of their questionnaire data for research, and for ongoing linkage to nationally held registry and health data. The study has Multi-Centre Research Ethics Committee approval (MREC 97/01).

Information about personal characteristics including height and weight, reproductive history, medical history, family history, menopausal status, and HT use was collected at baseline. The recruitment and follow-up questionnaires can be viewed at www.millionwomenstudy.org.

Postmenopausal women were defined as those who reported having undergone a natural menopause or having had a bilateral oophorectomy prior to recruitment. Body mass index (kg/m^2^) was calculated from self-reported weight and height at recruitment. This has been shown in Million Women Study participants to correlate closely with values based on measured variables [15]. Standard World Health Organization definitions [16] were used to categorise women with a BMI of <25 kg/m^2^ as ‘normal’, 25–29.9 kg/m^2^ as ‘overweight’, and 30 kg/m^2^ or more as ‘obese’. HT use was self-reported by participants at baseline. Reliability of reporting has been checked on a subset of participants and found to be excellent when compared with general practice prescription records [17].

All study participants are flagged on the NHS Central Registers, so that cancer registrations and deaths are routinely notified to study investigators. In addition, participants are linked using their name, date of birth, and NHS number (a unique personal identifier on all NHS health records) to NHS hospital admission databases, Hospital Episode Statistics (HES) in England and the Scottish Morbidity Records (SMR) in Scotland [18, 19]. The hospital records include day case and overnight stays to all NHS hospitals from April 1997 to March 31, 2011 (England); and from January 1981 to December 31, 2008 (Scotland). For each hospital admission, diagnoses are coded according to the International Classification of Diseases 10th revision (ICD-10) [20] and procedures coded according to the Office of Population Censuses Classification of Surgical Operations and Procedures (OPCS-4) [21].

The outcome of interest (‘surgically-confirmed fibroids’) is defined as a first hospital admission (including day-case admissions) after recruitment into the study with a primary diagnosis of uterine fibroids (ICD-10 D25) together with a related surgical procedure during the admission, limited to one (or more) of the following: dilation of cervix uteri and curettage of uterus (‘D&C’, OPCS-4 code Q10); diagnostic endoscopic examination of uterus (Q18); abdominal hysterectomy (Q074); vaginal hysterectomy (Q089); endoscopic resection of lesion of uterus (Q171); open myomectomy (Q092); open excision of lesion of uterus (Q093); vaginal excision of lesion of uterus (Q161); or diagnostic laparoscopy (T43).

Women who reported a natural menopause or bilateral oophorectomy at recruitment were included in the main analysis. Exclusion criteria included self-reported hysterectomy at baseline, hospital diagnosis of fibroids or record of hysterectomy prior to recruitment. Pre- or peri-menopausal women, those with indeterminate menopausal status, and those who began using HT prior to menopause were also excluded, as were those with missing information on height, weight, HT use or recruitment date.

We conducted an additional analysis looking at rates of surgically-confirmed fibroids in women who reported being pre-menopausal or peri-menopausal at recruitment, in order to compare them with postmenopausal women of the same age (50–54). These results are presented separately.

### Statistical analysis

Each participant contributed person-years to the analysis from the date of recruitment until the first identified end-point: date of hospital admission with surgically-confirmed fibroids, hysterectomy, date of death, emigration, or the end of the follow-up period. For women recruited in England follow-up was to 31 March 2011, and for women in Scotland to 31 December 2008. For the small proportion of women (5 %) recruited in England before 1 April 1997, person-years were calculated from this date. Earlier HES data, which was available in England from 1989, does not carry the individual’s NHS number thus we were only able to link to records from 1997 onwards.

Multivariate Cox regression models, with attained age as the underlying time variable, were used to estimate the relative risk of surgically-confirmed fibroids associated with BMI and HT use. Analyses were stratified for recruitment region (10 geographic regions corresponding to the areas covered by the cancer registries), and adjusted for deprivation using Townsend Deprivation Index quintiles [7], smoking (never, past, current), oral contraceptive use (never/ever), parity (0/1/2/3+), BMI (<25, 25–29, 30+), and HT use (never vs. ever). Missing values were included in the analysis as a separate category for each adjustment variable. Analyses were performed using Stata version 13 (Statacorp, 2013).

## Results

610,604 participants in the Million Women Study were postmenopausal, had not had a prior hysterectomy or recorded diagnosis of fibroids at recruitment, and provided information on their height, weight and HT use. 34 % were ever users of menopausal hormone therapy at baseline. 47 % were of normal weight, 36 % were overweight and 17 % were obese. Obese women were less likely to smoke, use HT, take regular vigorous exercise, or to come from the highest socioeconomic groups (Table 1).

**Table 1:** Participant characteristics and follow-up, by body mass index (BMI)

Characteristic	All women	BMI		
		<25 kg/m^2^ ‘Normal’	25.0–29.9 kg/m^2^ ‘Overweight’	≥30 kg/m^2^ ‘Obese’
Number of women (%)	610,604 (100.0)	287,208 (47.0)	217,546 (35.6)	105,850 (17.3)
Mean age at recruitment (SD)	57.8 (4.7)	57.6 (4.8)	58.0 (4.6)	57.9 (4.5)
Mean age at menarche (SD)	13.1 (1.6)	13.2 (1.6)	13.0 (1.6)	12.7 (1.6)
Past use of oral contraceptive %	52.2 (316,194)	54.5 (155,394)	50.9 (109,948)	48.5 (50,852)
Mean number of full term pregnancies (SD)	2.1 (1.3)	2.0 (1.2)	2.2 (1.3)	2.3 (1.4)
Nulliparous (n %)	12.3 (75,103)	13.6 (39,107)	11.0 (23,982)	11.4 (12,014)
Current smoker (n %)	20.4 (117,234)	22.8 (61,944)	19.1 (39,012)	16.5 (16,278)
Mean alcohol intake, units/week (SD)	3.9 (5.2)	4.3 (5.4)	3.8 (5.1)	2.8 (4.6)
Ever user of HT (n %)	34.0 (207,634)	36.3 (104,338)	33.5 (72,889)	28.7 (30,407)
Lowest quintile of socioeconomic status (n %)	26.3 (121,477)	23.6 (49,064)	26.5 (44,050)	33.0 (28,363)
Vigorous physical exercise at least once a week (n %)	38.6 (227,161)	44.1 (122,443)	36.9 (77,301)	27.0 (27,417)

Follow-up	All women	<25 kg/m^2^ ‘Normal’	25.0–29.9 kg/m^2^ ‘Overweight’	≥30 kg/m^2^ ‘Obese’
Woman-years (1000s)	6974	3302	2484	1188
Mean length of follow-up (SD)	11.4 (2.5)	11.5 (2.5)	11.4 (2.5)	11.2 (2.6)
Number of women with surgically-confirmed fibroids	3561	1508	1273	780

During 7 million person-years of follow-up, on average 11.4 years per woman, 3561 postmenopausal participants were admitted to hospital with a primary diagnosis of fibroids and a related surgical intervention.

**Fig. 1:**
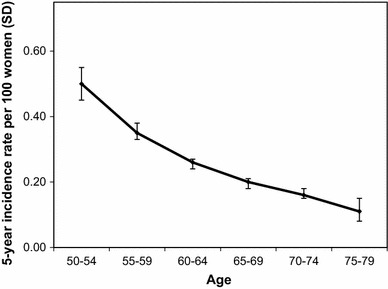
Five-year incidence rates per 100 women of surgically-confirmed fibroids in postmenopausal women aged 50–79, by 5-year age group

**Fig. 2:**
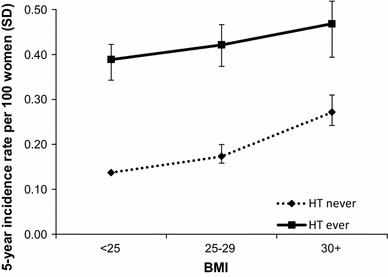
Five-year incidence rates per 100 women of surgically-confirmed fibroids by body mass index and menopausal hormone therapy use

Overall, the 5-year incidence rate of surgically-confirmed fibroids in the cohort was 0.3 %, or around 1 in 300 participants. This rate fell with age (Fig. 1), from 0.50 admissions per 100 women (0.50 %, 95 % CI 0.45–0.0.55) at ages 50–54, to 0.11 (95 % CI 0.08–0.15) in women aged 75–79. Women who reported never using HT had 5-year incidence rates which increased from 0.1 % (around 1 in 1000) in normal weight women to 0.3 % (1 in 300) in obese women, a three-fold increase (Fig. 2). Higher rates were seen in all BMI groups in women who reported ever using HT, giving an overall rate of 0.4 %, (or 1 in 250) over 5 years, with a smaller increase in rate with increasing adiposity compared with that seen in never users of HT.

At age 50–54, premenopausal women had a 5-year incidence rate of surgically-confirmed fibroids of 1.94 % (95 % CI 1.85–2.02), which was about four times that seen in postmenopausal women of the same age (0.50 %, 95 % CI 0.45–0.55).

Just over half (n = 1810, 51 %) of the women with surgically-confirmed fibroids had an abdominal or vaginal hysterectomy during their admission. A small number had another open procedure: myomectomy/excision of uterine lesion (n = 30, 1 %), and 7 % had a diagnostic laparoscopy (n = 244). The remainder had less extensive procedures, most commonly hysteroscopy, dilatation and curettage, or vaginal or endoscopic resection of uterine lesion.

Ever-use of HT increased the risk of surgically-confirmed uterine fibroids (Table 2). Among women who reported ever using menopausal hormone therapy, the relative risk was 2.33 (95 % CI 2.18–2.49), compared with never users, adjusted for smoking, oral contraceptive use, parity and deprivation.

**Table 2:** Adjusted^a^ relative risks of surgically-confirmed fibroids by menopausal hormone therapy (HT) use (ever vs. never)

HT use	Number of women	Number with fibroids	Adjusted RR (95 % CI)
Never	402,970	1607	1.00 (Reference group)
Ever	207,634	1954	2.33 (2.18–2.49)

^**a**^Adjusted for smoking, oral contraceptive use, parity and deprivation, stratified by region

Body mass index also had a significant impact on risk (Table 3). Compared with women of normal weight, those who were overweight (BMI 25–29.9) had a RR of 1.15 (95 % CI 1.07–1.24), and those who were obese (BMI 30+) a RR of 1.46 (95 % CI 1.33–1.59), adjusted for smoking, oral contraceptive use, parity and deprivation.

**Table 3:** Adjusted^a^ relative risks of surgically-confirmed fibroids by body mass index (BMI)

BMI	Number of women	Number with fibroids	Adjusted RR (95 % CI)
<25	287,208	1508	1.00 (Reference group)
25–29.9	217,546	1273	1.15 (1.07–1.24)
30+	105,850	780	1.46 (1.33–1.59)

^a^Adjusted for smoking, oral contraceptive use, parity and deprivation, stratified by region

While both HT use and BMI significantly increased risk of surgically-confirmed fibroids in postmenopausal women, when we looked at the interaction between the two risk factors, it was clear that BMI had a stronger effect in never-users of HT than in those who reported use of HT (Table 4). In never users of HT, there was a doubling of risk associated with obesity, RR 2.00 (95 % CI 1.77–2.26) compared with that seen in normal weight women. In women who reported ever having used HT, the absolute risks overall were much higher, although the increase in relative risk associated with rising BMI was smaller. The additional increase in risk associated with adiposity was still present, but the magnitude of this effect was smaller in women who used HT.

**Table 4:** Adjusted^a^ relative risks of surgically-confirmed fibroids by hormone therapy use and body mass index

Menopausal hormone therapy use	BMI	Number of women	Number with fibroids	Adjusted RR (95 % CI)
Never	<25	182,870	576	1.00 (Reference group)
	25–29.9	144,657	572	1.30 (1.16–1.46)
	30+	75,443	459	2.00 (1.77–2.26)
Ever	<25	103,338	932	2.78 (2.50–3.08)
	25–29.9	72,889	701	3.05 (2.73–3.41)
	30+	30,407	321	3.30 (2.86–3.79)

^a^Adjusted for smoking, oral contraceptive use, parity and deprivation, stratified by region

The fully adjusted relative risk of fibroids in ever users of HT of normal BMI was 2.78 (95 % CI 2.50–3.08), which rose to 3.05 (95 % CI 2.73–3.41) in the overweight group, and further to 3.30 (95 % CI 2.86–3.79) in women who were obese (Table 4).

## Discussion

We examined the relationship between adiposity, menopausal hormone therapy and the risk of surgically-confirmed uterine fibroids in postmenopausal women amongst participants in a large UK prospective cohort study. Use of HT doubled the risk of incident surgically-confirmed uterine fibroids in postmenopausal women at any BMI. Adiposity alone had a lesser, but still significant effect, with a 46 % increase in risk of fibroids in obese women irrespective of history of HT use. When stratified by HT use, increasing adiposity had a much stronger effect in women who had never used HT, with a doubling of risk in the obese group. The effect of BMI was also present in ever users of HT, but they had much higher risks at any BMI, and the additional effect of increased adiposity was smaller.

Other groups have reported that uterine fibroid risk is increased in overweight and obese women, although published findings are inconsistent. The majority of studies show an increasing risk with increasing BMI [11, 22–24] but some have reported a J-shaped association [25–27], no association [28–30], or even a decreased risk in obese women [31]. Two other cohort studies, the Nurses’ Health Study II [25] and the Black Women’s Health Study [27] used surgically-defined cases, and both found strong associations between BMI and the risk of fibroids, which our findings support.

There has been comparatively little published on the effect of HT on uterine fibroid risk. Several small studies with short-term follow-up have been published, and results have been contradictory. For example, Yang et al. [32] followed 37 early postmenopausal HT users and 35 matched controls who did not receive HT. All participants had a solitary uterine fibroid at recruitment. Fibroids were measured by transvaginal ultrasound at baseline and then annually for 3 years. In the 3rd year there was a significant increase in the fibroid volume in the HT group, but not in the control group. The women treated with HT were also significantly more likely to have an increase in fibroid volume of >25 %, however numbers were small. A review by Parker [10] which included Yang’s study, concluded that despite being hormone-sensitive tumours, for the majority of postmenopausal women with fibroids hormone therapy did not stimulate uterine or fibroid growth.

Two previous epidemiological studies that have examined the association between fibroids and HT use both reported increased risks. In a retrospective case–control study, Reed et al. [33] reported that use of combined oestrogen–progestagen HT was associated with an increased risk of fibroids, although the excess risk was largely confined to women who were not overweight (with BMI <24 kg/m^2^). In the California Teachers Study [34] women taking HT had a higher risk of surgically treated fibroids than women who had never used HT. An increased risk was seen both in women who used oestrogens alone (RR = 2.03, 95 % CI 1.17–3.52), and in those using combined HT (RR = 2.38, 95 % CI 1.66–3.41), similar to our overall findings.

Ovarian hormones are thought to play a key role in the aetiology of fibroids. Early menarche and obesity increase the incidence of fibroids, whereas high parity appears protective [35]. Fibroids are hormone-sensitive tumours, and so it is perhaps not surprising that use of HT doubles incidence rates in postmenopausal women. Adiposity is thought to affect the risk of fibroids in postmenopausal women because peripheral tissues, principally body fat, become the major source of circulating oestrogens (when women are not taking HT) [36].

Our results suggest that fibroids do continue to be an issue in postmenopausal women. Rates fall with age, and in women 50–54, the only age group which includes substantial numbers of both premenopausal and postmenopausal women, menopausal status affects incidence. At all ages, uterine fibroids occur more frequently in postmenopausal women who are obese, and those who use HT. Data in our study were collected prospectively, and we limited our analysis to women with a primary diagnosis of uterine fibroids during their hospital admission who also had a related operative procedure. Our aim was to identify clinically significant fibroids associated with a surgical intervention. A limitation of our study is that we were not able to assess whether having a raised BMI or using HT increased the risk of developing new fibroids in the postmenopausal period, or whether these factors decrease the likelihood of regression or encourage growth of pre-existing fibroids.

What is the public health relevance of our findings? Over half of the postmenopausal women with surgically-confirmed fibroids in our cohort underwent a hysterectomy or other major abdominal surgical procedure associated with their disease. Approximately 50,000 hysterectomies are performed annually in the UK [37]. At around £3282 per case [38], the 1810 hysterectomies in our study alone would have cost the NHS about £6 million. The cost in terms of quality of life, lost earnings, and morbidity to the women may have been even greater. Surgically-confirmed fibroids continue to occur in postmenopausal women, especially those who are obese or using menopausal hormone therapy. HT and BMI are potentially modifiable risk factors for uterine fibroids in postmenopausal women.
